# Absence vs. presence

**DOI:** 10.1186/1865-1380-7-S1-O6

**Published:** 2014-07-25

**Authors:** Swaminathane Ramkumar

**Affiliations:** 1 EMS Memorial Co-operative Hospital, Perinthalmanna, Kerala, India

## Objective

To study the psychological outcomes of bystanders after witnessing CPR in the resuscitation bay.

## Methods

This is a prospective & observational study. The total sample (120) was divided into two groups, Test (60) and Control (60). Both Test & Control group (Witnessed & Unwitnessed) were contacted over telephone, their psychological state was assessed using a standard stress assessment scale, The Impact of Event Scale (IES) by M.Horowitz *et al*. A structured proforma was prepared to record the data. Details, and willingness of the bystander to witness, age, gender and, contact details of the bystander, unpleasant reactions of the bystanders while witnessing, outcome of resuscitation were recorded. 30 days after the incident, the bystander was contacted over telephone, their psychological state was assessed. 

## Results

Of the 120 cardiac arrests that were witnessed, 91 (75.8%) were pre hospital and bystanders willingness to help 110 (91%), and 3 (2.5%) bystanders had an adverse outcome. The outcomes included, dizziness, running away from the scene and crying out loud. In test group around 92% experienced mild & subclinical psychological impact, out of which 70% (42) were in subclinical range, 3% (2) had medium range of psychological impact. 5% (3) could not be reached over phone even after trying for 10 times. In control group 20% (4-subclinical, 8-mild range) and the remainder were below the cut off for subclinical. Bystander presence during CPR did not adversely affect the mental state of the witnessing person much. The psychological outcome in a person who has not witnessed is not as good as when compared to that of who have witnessed the CPR.

## Limitations

Not a multicenter study.

## Conclusion

Based on the results in this study it is postulated that family presence during CPR is not adversely affecting the witnessing person much. The psychological outcome in a person who has not witnessed is not as good when compared to that of who have witnessed the CPR. It is better to give a choice to a relative to witness the CPR and be near their dear one during CPR in the resuscitation bay.

